# Perioperative estimations of oxygen consumption from LiDCO™plus-derived cardiac output and Ca-cvO2 difference: Relationship with measurements by indirect calorimetry in elderly patients undergoing major abdominal surgery

**DOI:** 10.1371/journal.pone.0272239

**Published:** 2024-07-25

**Authors:** Julia Jakobsson, Carl Norén, Eva Hagel, Magnus Backheden, Sigridur Kalman, Erzsébet Bartha

**Affiliations:** 1 Division of Anaesthesia and Intensive Care, Department of Clinical Science, Intervention and Technology (CLINTEC), Karolinska Institutet, Stockholm, Sweden; 2 Department of Perioperative Medicine and Intensive Care, Karolinska University Hospital Huddinge, Stockholm, Sweden; 3 Department of Anaesthesia and Intensive Care, Nyköping County Hospital, Nyköping, Sweden; 4 Department of Learning, Information, Management and Ethics (LIME), Medical Statistics Unit, Karolinska Institutet, Stockholm, Sweden; Aristotle University of Thessaloniki School of Veterinary Medicine, GREECE

## Abstract

**Background:**

Feasible estimations of perioperative changes in oxygen consumption (VO_2_) could enable larger studies of its role in postoperative outcomes. Current methods, either by reverse Fick calculations using pulmonary artery catheterisation or metabolic by breathing gas analysis, are often deemed too invasive or technically requiring. In addition, reverse Fick calculations report generally lower values of oxygen consumption.

**Methods:**

We investigated the relationship between perioperative estimations of VO_2_ (EVO_2_), from LiDCO^™^plus-derived (LiDCO Ltd, Cambridge, UK) cardiac output and arterial-central venous oxygen content difference (Ca-cvO_2_), with indirect calorimetry (GVO_2_) by QuarkRMR (COSMED srl. Italy), using data collected 2017–2018 during a prospective observational study on perioperative oxygen transport in 20 patients >65 years during epidural and general anaesthesia for open pancreatic or liver resection surgery. Eighty-five simultaneous intra- and postoperative measurements at different perioperative stages were analysed for prediction, parallelity and by traditional agreement assessment.

**Results:**

Unadjusted bias between GVO_2_ and EVO_2_ indexed for body surface area was 26 (95% CI 20 to 32) with limits of agreement (1.96SD) of -32 to 85 ml min^−1^m^−2^. Correlation adjusted for the bias was moderate, intraclass coefficient(A,1) 0.51(95% CI 0.34 to 0.65) [F (84,84) = 3.07, *P*<0.001]. There was an overall association between GVO_2_ and EVO_2_, in a random coefficient model [GVO_2_ = 73(95% CI 62 to 83) + 0.45(95% CI 0.29 to 0.61) EVO_2_ ml min^−1^m^−2^, *P*<0.0001]. GVO_2_ and EVO_2_ changed in parallel intra- and postoperatively when normalised to their respective overall means.

**Conclusion:**

Based on this data, estimations from LiDCO^™^plus-derived cardiac output and Ca-cvO_2_ are not reliable as a surrogate for perioperative VO_2_. Results were in line with previous studies comparing Fick-based and metabolic measurements but limited by variability of data and possible underpowering. The parallelity at different perioperative stages and the prediction model can provide useful guidance and methodological tools for future studies on similar methods in larger samples.

## Introduction

A postoperative imbalance between oxygen consumption and delivery, leading to increased oxygen extraction, has been associated with increased morbidity and mortality after major surgery [1]. The focus of goal-directed haemodynamic therapy (GDHT) has traditionally been on oxygen delivery, which is often easier to assess and to develop measurable optimisation strategies for [2]. Recently, interest is growing to reassess perioperative oxygen consumption in current surgical populations using modern monitoring and analytic methodologies [3–6]. Feasible estimations could enable larger studies on the role of oxygen consumption in postoperative outcomes. Available techniques, by pulmonary artery catheterisation or indirect calorimetry, are either deemed too invasive or difficult to manage in a clinical study setting during non-cardiac surgery. Using oxygen uptake calculated from fractions of inspiratory and expiratory oxygen in the closed breathing circuit during low-flow anaesthesia [7] has not demonstrated agreement when compared to standard methods [8]. Importantly, it can not be used in awake patients in the postoperative period. Commonly used haemodynamic monitoring in major surgery, such as minimal-invasive cardiac output with arterial and central venous access, could offer a possibility not only to estimate intra- and postoperative oxygen consumption but also to follow changes over time. By substituting mixed with central venous oxygen content and using the cardiac output derived from a minimal-invasive monitor, an estimation of oxygen consumption could theoretically be calculated by the reverse Fick principle [9]. The lack of absolute agreement between calorimetric and Fick-based methods has been reported previously, the latter do not include pulmonary oxygen consumption and global oxygen consumption values are usually reported around 20–40 ml min^−1^m^−2^ lower compared to those obtained from breathing gas analysis [10–12]. Examples of previous studies comparing methods for assessing oxygen consumption by either breathing gas analysis or Fick-based measurements are presented in Table 1. Yet, if this bias remains unchanged in the intra- and postoperative period, such estimations could be studied in larger samples and related to other clinical parameters and outcomes.

**Table 1:** Examples of previous studies comparing methods for gas-derived VO2 with Fick-derived VO2.

Author, year	Subjects, N =	Number of paired measurements	Method gas-derived VO_2_	Method Fick-derived VO_2_	Bias (SD or 95% CI), limits of agreement in ml min^-1^ m^-2^	Statistical methodology
Bizouarn et al. 1992 [17]	Postop cardiac surgery, N = 10	50	IC Deltatrac^®^	PAC thermodilution	34 (SD 27) LoA: -33 to 88	B-A ANOVA for time-effects
Bizouarn et al. 1995 [25]	Postop cardiac surgery, N = 9	54	IC Deltatrac^®^	PAC (continuous thermodilution)	15 (95% CI, 13 to 17) LoA: -3 to 33	B-A PE-RE
Epstein et al. 2000 [26]	Trauma ICU, N = 38	152	IC Puritan Bennett^®^	PAC thermodilution	41 (95% CI, 20 to 63) LoA*: lower -40 to -72, upper 120 to 149	B-A
Hofland et al. 2003 [18]	Intraop vascular surgery, N = 11	73	CC Physioflex^®^	PAC thermodilution	36 (not presented) LoA**: -40 to 112	B-A Linear regression/Spearman rank correlation
Inadomi et al. 2008 [20]	Postop major abdominal surgery, N = 28	56	IC Puritan Bennett^®^	CVC+PDD	33 (not presented) LoA: -31 to 97	B-A Linear regression
Keinanen and Takala, 1997 [10]	Periop cardiac surgery, N = 9	45	IC Deltatrac^®^	PAC thermodilution	33 (25) LoA (not analysed)	Linear regression ANOVA
Leonard et al. 2002 [8]	Periop cardiac surgery, N = 29	29***	CC Biro method	PAC thermodilution	75 (121) LoA: -162 to 311	B-A
Myles et al. 1996 [27]/revised 2007 [19]	Periop cardiac surgery, N = 20	143	IC Deltatrac^®^	PAC thermodilution	20 (50) LoA -128 to 88, revised 30 (-116 to 57)	B-A (1996) Random effects model (2007)
Peyton and Robinson, 2005 [11]	Intraop cardiac surgery, N = 9	18	Modified Bains circuit	PAC thermodilution	19 (20) (95% CI, 9 to 29) ml min^-1^ LoA (not analysed)	Mean difference
Saito et al. 2007 [12]	Periop oesophag-ectomy, N = 35	210	IC Deltatrac^®^	PAC thermodilution	23 (95% CI, 20 to 27) LoA: -23 to 69	B-A Correlation Difference over time
Smithies et al. 1991 [28]	General ICU, N = 8	20	CC spirometry	PAC thermodilution	36 (SD29) ml min^-1^ LoA (not analysed)	Mean difference
Soussi et al. 2017 [29]	ICU burns patients, N = 22	44	IC E-COVX^®^	CVC +PiCCO^®^	60 (not presented) LoA: -84 to 203	Linear regression Bland-Altman
Stuart-Andrews et al. 2007 [30]	Intraop cardiac surgery, N = 30	30***	Modified semi-closed breathing circuit	PAC thermodilution	21 (25) LoA (overall in graph)	Correlation Bland-Altman
Walsh et al. 1998 [31]	ICU hepatic failure, N = 17	98	IC Deltatrac^®^	PAC thermodilution	-41(30) (95% CI, -31 to -47) LoA: -101 to 19 (Fick–Gas)	Bland-Altman Repeatability

Abbreviations: IC; indirect calorimetry: PAC; pulmonary artery catheter: B-A; Bland-Altman method for assessing agreement: ANOVA; analysis of variance; PE: percentage error; RE: relative error: ICU; intensive care unit: CC; closed circuit anaesthesia system: CVC; central venous catheter: PDD; pulse dye densitometry:

* no overall LoA.

** derived from graph

***pre-CPB measurements.

We aimed to investigate the relationship and temporal changes between estimations of oxygen consumption (EVO_2_), from LiDCO™plus-derived cardiac output and blood gas sampling from arterial and central venous lines, and measured oxygen consumption (GVO_2_). We used prospectively collected data from an observational study on perioperative oxygen consumption and delivery in elderly patients undergoing major abdominal surgery [13].

## Materials and methods

This was a pre-planned prospective explorative study based on data collected during an observational study in patients > 65 yrs undergoing open liver or pancreatic surgery between Dec 2017 and April 2018 (clinicaltrials.gov NCT03355118). The results on oxygen transport parameters from that study has been published [13]. The Regional Ethics Review Board of the Stockholm Region (ID 2017/291-31/4) approved the study and written informed consent was obtained from all participants.

### Patients and settings

A description of selection and enrolment, patient characteristics’ and perioperative management can be found in the previous publication [13]. As stated there, 20 ASA II-IV patients over 65 years undergoing open pancreatic or liver resection surgery in epidural and general anaesthesia were included. The study was conducted at the Karolinska University Hospital in Huddinge, a tertiary referral center for upper abdominal surgery.

### Data extraction and time-points

Paired values of oxygen consumption by estimations based on cardiac output monitoring from LiDCO™plus and arterial-central venous blood gas samples (EVO_2_) and indirect calorimetry GVO_2_) from five perioperative time-points were analysed; T1: during anaesthesia, right before surgical skin incision; T2: early during surgery, directly after skin incision; T3: later during surgery, >2h after skin incision; T4: early postoperatively, <12h after extubation; T5: late postoperatively, on postoperative day 1. The mean values for GVO_2_ during the approximate 20-minute measurement periods were compared with simultaneous cardiac output measurements averaged for each minute exported from LiDCOviewPRO (LiDCO Ltd, Cambridge, UK). The blood gas parameters were calculated as means of two simultaneously drawn arterial and central venous samples at 5 and 15 minutes into the measurement period.

### Measurements of VO2 by indirect calorimetry (GVO2)

Indirect calorimetry was performed by QuarkRMR (COSMED srl, Italy). This device applies a breath-by-breath technique to measure gas flow and concentrations that are synchronised by data processing algorithms. The Haldane transformation is used to calculate oxygen consumption [14]. During intraoperative measurements, the flow meter (Flow-REE, COSMED srl, Italy), gas sampling line and moist filter were placed between the endotracheal tube and the Y-piece of the ventilator. The ventilator was set to a fresh gas flow of 2 L min^-1^ and FiO_2_ of 0.5 during measurements to allow for gas sampling. All other ventilation settings were left unchanged. Postoperative measurements were made with a tight-fitting face mask connected to a bidirectional turbine flow meter and a gas sampling line. No supplemental oxygen was administered during the postoperative measurements. The calorimeter was calibrated before start of intraoperative measurements and before each postoperative measurement after a warm-up time of 20 minutes with a standardised gas mixture containing 16% oxygen and 5% carbon dioxide. The gas sampling line, Flow-REE and moist filter were changed before each measurement (except before T2, continuous to T1) and all flowmeters were calibrated with a 3L-syringe.

### Estimation of VO2 by minimal-invasive cardiac output and arterial-central venous oxygen content difference (EVO2)

EVO_2_ was calculated by the reverse Fick’s principle with central venous instead of pulmonary artery blood using the following formulas: [15]

$${\text{EVO}}_{2}=CO\times {\text{Ca-cvO}}_{2}\;\text{x}\;10$$

$${\text{Ca-cvO}}_{2}=\text{Hb}\times 1.31\times ({\text{SaO}}_{2}-{\text{ScvO}}_{2})+0.0225\times (P{\text{aO}}_{2}-P{\text{cvO}}_{2})$$

[*CO*; cardiac output in L min^-1^, Ca-cvO_2_; oxygen content difference between arterial and central venous blood in ml dl^-1^, Hb; haemoglobin in g dl^-1^, SaO_2_; arterial oxygen saturation, ScvO_2_; central venous saturation, *P*aO_2_; partial pressure of oxygen in arterial blood, *P*cvO_2_; partial pressure of oxygen in central venous blood, constants 1.31 and 0.0225, referring to the Hüfner constant and the solubility coefficient of oxygen (ml O_2_ dl^-1^ kPa^-1^), and 10 as a conversion factor from dL to L.].

Cardiac output was obtained from LiDCO^™^plus (LiDCO Ltd, Cambridge, UK). The device was calibrated and recalibrated a minimum of three times according to the manufacturer’s instructions. This was done using a transpulmonary lithium bolus indicator dilution technique for an absolute CO value to obtain a calibration factor and to perform autocalibration after which continuous measurement of haemodynamic variables is carried out by the pulse power analyses integrated in the LiDCO^™^plus system. Calibration procedures was undertaken at times to avoid interference by non-depolarising muscle relaxants. Missing values from *CO* measurements (averaged for each minute) were substituted by linear interpolation between the subsequent measurements, making sure not more than three data points were missing, and no major haemodynamic changes occurred. Blood gases were analysed immediately after sampling by ABL800 Flex or ABL90 Flex (Radiometer Medical ApS, Denmark). Cardiac output and measured oxygen consumption were indexed for body surface area using the DuBois formula yielding values of GVO_2_ and EVO_2_ in ml min^−1^m^−2^ [16].

### Statistical analysis

The sample size calculation was performed for the primary study [13], based on a previous meta-analysis [4], from which 20 patients were expected to demonstrate a relevant change in oxygen consumption after induction of anaesthesia. This would yield a maximum of 100 paired measurements of EVO_2_ and GVO_2_ which was considered sufficient based on sample sizes in previous studies (Table 1) and with a possible >10% data loss. Continuous data was tested for normality distribution and statistical tests applied accordingly. Statistical analyses were performed and constructed in R (version 3.5.3; R Foundation for Statistical Computing, Vienna, Austria, URL; https://www.R-project.org) and SAS (version 9.4; SAS Institute Inc, Cary, NC, U.S.). The statisticians conducting the analyses were not involved in the data collection. Mean difference between EVO_2_ and GVO_2_ with 95% confidence interval were calculated from the individual paired measurements and grouped by time point (T1-5). These changes over time were analysed by linear mixed models with Holm-adjusted Tukey post-hoc tests. To investigate the overall association between EVO_2_ and GVO_2_, a random coefficient model was used based on individual slopes and coefficients. Analyses of the perioperative changes over time of GVO_2_ compared to EVO_2_ and its input variables (*CI*; cardiac index and Ca-cvO_2_) were conducted by random effect mixed models with method or component and time as fixed effects. Adjustment for differences in variances of the methods or components was made. In these models, the relative changes were normalised to the patients’ individual baseline measurements (T1). In the model analysing changes of each method in awake and anaesthetised subjects, the changes were normalised to the respective overall mean.

Traditional agreement assessment was also performed by intraclass correlation and Bland-Altman analysis. Single score intraclass correlation was used, a in a two-way model yielding ICC coefficients with 95% CI. Bias and limits of agreement with 95% CI was visualised in Bland-Altman plots. Both ICC and Bland-Altman analyses were performed separately for each time-point T1–T5. The overall ICC and Bland-Altman analyses were not adjusted for repeated measurements as these were performed under varying intra- and postoperative conditions. Normality and homoscedasticity were assessed in residual plots. An alpha of 0.05 was considered significant.

## Results

A total of 85 paired measurements of EVO_2_ (LiDCO™plus-derived cardiac output Ca-cvO2) and GVO_2_ (measurements by indirect calorimetry) were obtained in 20 subjects; 58 were obtained intraoperatively and 27 in the postoperative period. Four paired intraoperative measurements were not performed due to early termination of surgery (unexpected metastatic spread of malignancy) in two patients. Thirteen paired measurements could not be performed in the postoperative period because of technical or arterial line failure (n = 2), logistical reasons (n = 2), patients’ decline (n = 3), exclusion due to short postoperative stay (n = 4) and need for supplemental oxygen (n = 2). Correct positioning of the CVC was confirmed by postoperative chest x-ray in all patients. As stated above, patients’ characteristics and perioperative data along with enrolment details can be found in the main oxygen transport study [13].

**Fig 1:**
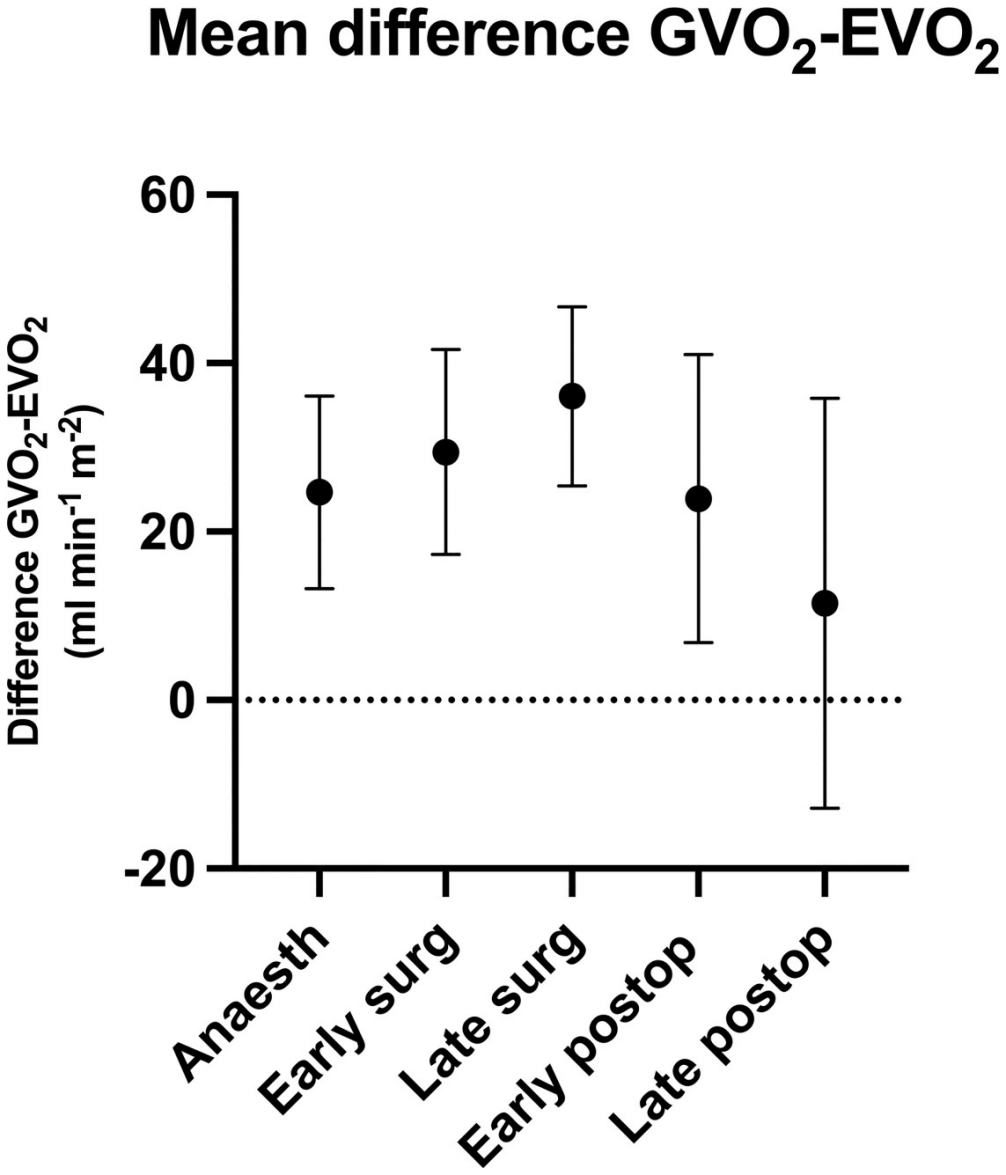
Difference between oxygen consumption measured indirect calorimetry (GVO_2_) and estimated from LiDCOplus™-derived cardiac output and Ca-cvO_2_ (EVO_2_) at each perioperative time-point; anaesthesia (before skin incision), early surgery (after skin incision), late surgery (>2hrs after skin incision), early postop (<12 hrs after extubation, late postop (postoperative day 1) expressed as mean (95% CI) ml min^-1^ m^-2^.

Taking all 85 paired measurements together, EVO_2_ was generally lower than GVO_2_ with an overall mean difference of oxygen consumption between EVO_2_ and GVO_2_ of -26 (95% CI -20 to -32; *P*<0.001) ml min^−1^m^−2^. The difference at the different perioperative stages (anaesthesia, early and late surgery, early and late postoperative) is presented in Fig 1. The changes between these stages were not statistically significant. [F(4, 168) = 1.39, *P* = 0.241]. The mean overall difference between GVO_2_ and EVO_2_ unadjusted for body surface area was -50 (95% CI -61 to -39; *P*<0.001) ml min^-1^. Percentage error (PE) for all measurements was 30 (95% CI 26 to 34) % with a coefficient of variation of 61%. Intraoperative measurements had a PE of 34 (95% CI 30 to 38) % and postoperative 21 (95% CI 13 to 29) % with coefficients of variation of 47% and 94%, respectively.

**Fig 2:**
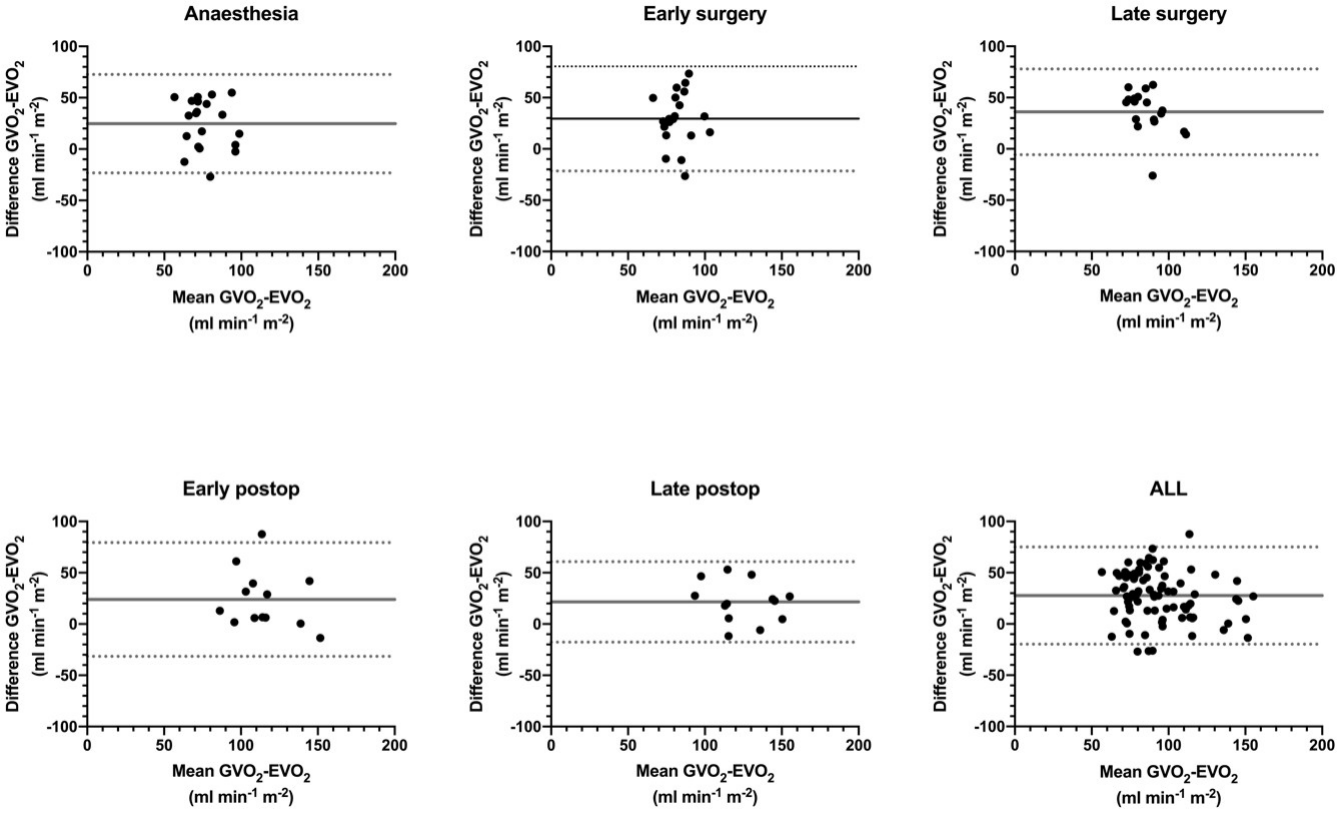
Bland-Altman plots describing mean GVO_2_-EVO_2_ vs ΔGVO_2_-EVO_2_ in ml min^-1^ m^-2^ at the different time-points with bias (continuous lines) and limits of agreement (dotted lines). Anaesthesia (T1; N = 20); b. Early surgery (T2; N = 20); c. Late surgery (T3; N = 18); d. Early postop (T4; N = 13); e. Late postop (T5; N = 14); f. all time-points in the same plot (N = 85 paired measurements).

Bland-Altman plots were constructed to illustrate the bias and limits of agreement between EVO_2_ and GVO_2_ at the different time-points, see Fig 2. The overall unadjusted mean bias was 26 ml min^-1^ m^-2^ with limits of agreement (1.96SD) of -32 to 85 ml min^−1^m^−2^. Excluding one outlier in the late postoperative period (a patient with a large Ca-cvO_2_ difference) changed the unadjusted bias to 28 (LoA -20 to 75) ml min^-1^ m^-2^. The overall correlation for *absolute* agreement was poor, with an intraclass coefficient ICC(A,1) of 0.37 (95% CI 0.34 to 0.65) [F(84,10.2) = 3.07, *P* = 0.0266], and improved to moderate but with large confidence intervals when adjusted for lower overall mean difference of EVO_2_, ICC(A,1) = 0.51 (95% CI 0.34 to 0.65) [F(84, 84) = 3.07, *P*<0.001]. Graphs depicting the correlation between indexed GVO_2_ and EVO_2_ at the different time-points (T1–5) including the unadjusted overall correlation are presented in S1 File.

**Fig 3:**
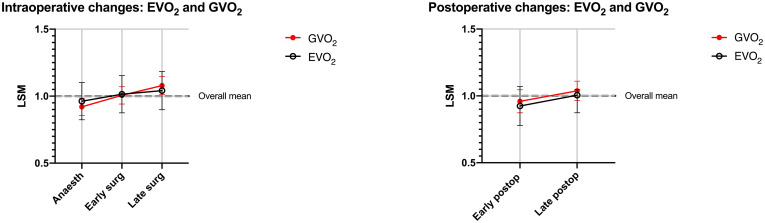
**a** and **b.** Perioperative changes of GVO_2_ (red) and EVO_2_ (black) separated for anaesthetised (a) and awake postoperative (a) states. Least square means estimates with 95% CI and normalised to overall means (= 1.0) of each method in anaesthetised intraoperative (T1–T3) and awake postoperative states (T4–T5). (T1) anaesthesia; Early surgery (T2); Late surgery (T3); Early postop (T4); Late postop (T5).

**Fig 4:**
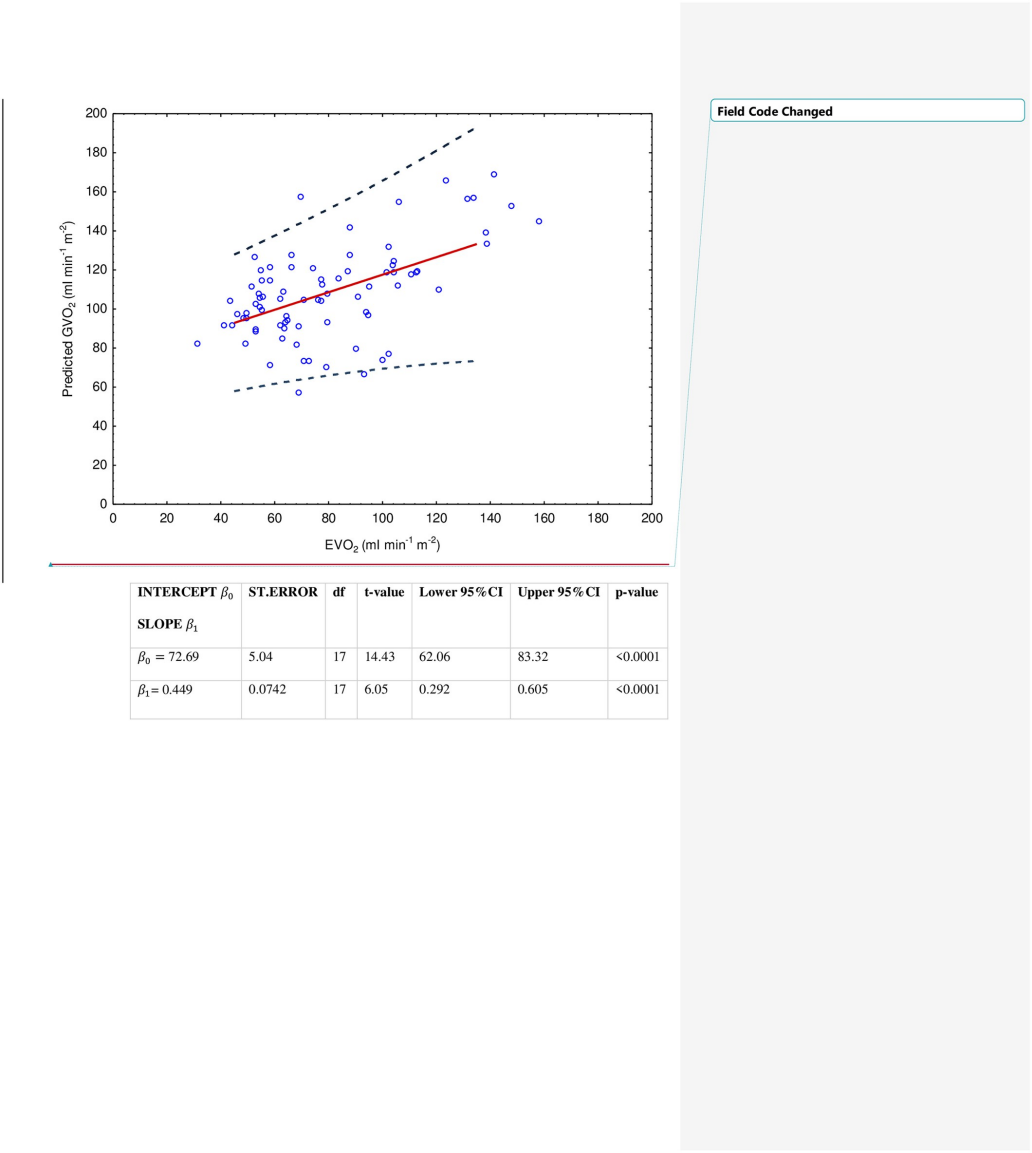
A random coefficient model for predicted GVO_2_ from EVO_2_ based on all perioperative time-points. **GVO**_**2**_ **= β0 + β1 (EVO**_**2**_**).** Two patients with a total of only 2 paired intraoperative measurements(T1–T2) were excluded from the analysis.

Parallel changes were demonstrated between GVO_2_ and EVO_2_ when separated to the anaesthetised intraoperative state [F(2, 49.9) = 0.57, *P* = 0.5669] and the awake postoperative state F(1, 22) = 0.00, *P* = 0.9604), Fig 3a and 3b. An overall association between GVO_2_ and EVO_2_ was demonstrated in a random coefficient model for predicting GVO_2_ from EVO_2_, but with large predictions intervals as illustrated by the model coefficients (Fig 4). The two patients with early termination of surgery were excluded from this analysis. The variances of EVO_2_ and its components, oxygen content difference in arterial and central venous blood (Ca-cvO_2_) and cardiac index (*CI*) were larger compared to GVO_2_ at all time-points, these analyses are presented in S2 File.

## Discussion

To the best of our knowledge, this is the first study investigating a Fick-based estimation method for perioperative oxygen consumption based on commonly used haemodynamic monitoring (LiDCO^™^plus and blood gas sampling from arterial and central venous lines). Bias and agreement with indirect calorimetry were approximate to previous studies using pulmonary artery catheters in which Fick-based methods have demonstrated 20–40 ml min^-1^ m^-2^ lower values compared to metabolic measurements. As in many of these studies, limits of agreement and coefficients of variation were unacceptably large [12, 17–19]. The relationship between estimated and measured oxygen consumption was investigated by intraclass correlation, parallelity and with a random coefficient model. Parallelity and overall association was demonstrated, but the model had large prediction intervals, probably attributable to the limited number of observations and the variability of collected data. This estimation method cannot be recommended as clinically useful to assess perioperative oxygen consumption. Nevertheless, it can provide important guidance in the design and analytical approaches of future studies involving precise monitoring and larger number of observations.

Most previous studies investigating methods for oxygen consumption monitoring perioperatively or in critically ill patients were performed decades ago using traditional method comparison analytical methods. Examples of the earlier method comparison studies are summarised in Table 1. Newer studies using non-invasive cardiac output monitors have not shown agreement with oxygen consumption measurements from indirect calorimetry [20] or pulmonary artery catheters [21]. However, the monitors used were not calibrated by transpulmonary or indicator dilution such as the PiCCO^™^ or LiDCO^™^plus systems and did not analyse changes over time. Estimates of increased oxygen extraction, i.e low mixed or central venous saturation, are associated with poor surgical outcomes [22, 23]. However, cut-off levels remain unclear and the quality of evidence is low [24]. In order to further study and distinguish the role of oxygen consumption in the perioperative period, feasible estimations are needed. Oxygen consumption calculated by the reverse Fick equation is consistently reported lower than simultaneous measurements by analysis of respiratory gas exchange [8, 10–12, 17, 25–32]. This difference or bias has been attributed to the pulmonary oxygen consumption [30, 33, 34]. However, variability of Fick-derived measurements [28, 31] and wide limits of agreement [19] has made it difficult to estimate a systematic methodological bias. Many previous studies have either been performed in thoracic or cardiovascular surgery [10–12, 17, 33] or in critically ill patients [26, 29, 31, 35]. Pulmonary oxygen consumption can be expected to increase after thoracic surgery [12] and in intensive care patients with varying degrees of lung injury [36]. Some studies that involve patients undergoing predominately abdominal surgery have shown acceptable agreement between the methods [37, 38]. The age of the studies is also reflected by the frequent use of the Deltatrac Metabolic Monitor^®^ (Datex Instrumentarium, Helsinki, Finland), a metabolic monitor using a mixing-chamber technique and which is no longer in production. Many metabolic monitors in modern clinical use are based on breath-by-breath technology such as the Es-COVX^®^ (GE Healthcare, Helsinki, Finland) or the QuarkRMR^®^ in our study. Although there is supporting evidence for some overestimation of oxygen consumption, the technology has shown clinically acceptable agreement when compared with mixing-chamber methods [39, 40] and it has been validated in the semi-closed circle absorber systems commonly used in anaesthesia [41]. Our results on GVO_2_ were comparable with studies using Deltatrac II when corrected for difference in units (Table 1). The estimations of oxygen consumption rely on accurate cardiac output determinations and oxygen content difference measurements. The LiDCO^™^plus has shown acceptable performance against the pulmonary artery catheter and other devices in cross-comparisons in cardiac output accuracy studies [42, 43]. During rapidly changing haemodynamic situations, concerns regarding trending ability and underestimation of cardiac output have been raised [44, 45]. The 20-minute data extraction periods in this study were specifically chosen to represent perioperative time points that usually are without considerable circulatory instability. Central and mixed venous oxygen saturation have not shown interchangeability [46–48] but some studies have suggested that trends in ScvO_2_ can replace SvO_2_ [49–51]. During stable intraoperative conditions, oxygen content difference is not expected to vary to a large extent whereas cardiac output can show considerable in- and between patient variability [21]. In our study, oxygen content difference and cardiac output demonstrated similar coefficients of variation.

The known lack of agreement between gas- and Fick-derived measurements of oxygen consumption and the lack of a clinically available golden standard method led us to apply alternative analytical approaches. Time effects and repeated measurements in the same subject under changing conditions constitute important statistical challenges in studies involving perioperative patients. Previous studies have often used simple linear regression or correlation [10, 11, 20, 29] or Bland-Altman analysis [52] without correction for repeated measurements [12, 20, 26, 37] except for some [19, 32]. Only a few address the relationship between measurements over time [17, 27]. In the present study, we developed a prediction model for EVO_2_ and GVO_2_ by using a random coefficient model based on individual slopes and intercepts. A significant positive association was demonstrated here, but with large prediction intervals. Such prediction models should obviously be evaluated in larger samples. We also present analyses of relative changes of EVO_2_ and its components with GVO_2_. The parallelity that was demonstrated could indicate an ability of EVO_2_ to track changes in oxygen consumption. To address this further, multiple measurements during shorter periods of time would be required. Analytic models previously used for cardiac output monitors such as polar plot approaches could assess the magnitude and direction of changes [44]. Intraclass correlation (ICC) was used as it better reflects reliability and agreement based on analysis of variance of the pooled data [53]. When adjusted for the consistently lower values of EVO_2_, the ICC estimates of the model improved but not so much (ICC coefficient 0.51 vs 0.37). Bland-Altman analysis has since long been the standard method for visualisation of agreement when comparing different methods of oxygen consumption monitoring [19]. Myles and Cui further elaborated the methodological issues related to repeated measurements in the same subject already considered by Bland and Altman [54] and proposed different random effects models to adjust limits of agreement [19]. As measurements were performed under varying perioperative conditions, we present the time-points separately and did not adjust the overall limits of agreement for repeated measurements in the same patient.

This study has several major limitations in addition to those discussed above. Although the number of observations is in the same range as in many previous studies (Table 1), no sample size calculation was performed for the specific analytical approaches that were applied. Consequently, it is possibly underpowered for many of these outcomes. There was a considerable loss of data in the postoperative period limiting the conclusions on the changes at the different perioperative stages.

In summary, this estimation method cannot be regarded as clinically useful, and results were comparable to previous comparisons between Fick-based and metabolic methods. Large variability of data and possible underpowering limited the construction of a prediction model and determination of a precise systematic bias. The results on parallelity and the overall association between the methods should be regarded as indicative. That aside, it can provide useful methodological tools for future studies on oxygen consumption assessment.

## Supporting information

S1 File Intraclass correlation comparing GVO2 and EVO2 at different perioperative stages. S1. Two-way single score intraclass correlation, ICC (A,1), of GVO2 and EVO2 indexed for body surface area (i) in ml min-1 m-2 at the different time-points with ICC coefficients (95% CI): a. Anaesthesia (T1; N = 20) -0.066 (-0.187,0.273); b. Early surgery (T2; N = 20) -0.131 (-0.285, 0.212); c. Late surgery (T3; N = 18) 0.019 (-0.081, 0.212); d. Early postop (T4; N = 13) 0.214 (-0.164, 0.619) e. Late postop (T5; N = 14) 0.224 (-0.316, 0.660); f. overall (see text for details).
(TIF)

S2 File Parallelity analyses between GVO2 and EVO2 and its components. ***S2*:*1*** *Mixed effect models for parallelity analysis between GVO2 and EVO2 normalised to baseline Anaesthesia (T0) (= 1*.*0); Early surgery (T2); Late surgery (T3); Early postop (T4); Late postop (T5) pp*. *2–9* **S2:2.** *Mixed effect models for parallelity analysis between GVO2 and Ca-cvO2 (arterio-central venous oxygen content difference) normalised to baseline Anaesthesia (T0) (= 1*.*0); Early surgery (T2); Late surgery (T3); Early postop (T4); Late postop (T5) pp*.*10–17* **S2:3** *Mixed effect models for paralellity analysis between GVO2 and CI (cardiac index) normalised to baseline Anaesthesia (T0) (= 1*.*0); Early surgery (T2); Late surgery (T3); Early postop (T4); Late postop (T5) pp*.*18–25* **S2:4.***Mixed effect models for parallelity analysis between GVO2 and EVO2 normalised to overall mean for each method (= 1*.*0) in anaesthetized subjects Anaesthesia (T0); Early surgery (T2); Late surgery (T3) pp*.*26–31***S2:5.** *Mixed effect models for paralellity analysis between GVO2 and EVO2 normalised to overall mean for each method (= 1*.*0) in awake subjects Early postop (T4); Late postop (T5) pp*. *32–37*.
(DOCX)
