# Supplementary figures and images for: Perioperative estimations of oxygen consumption from LiDCO™plus-derived cardiac output and Ca-cvO2 difference: Relationship with measurements by indirect calorimetry in elderly patients undergoing major abdominal surgery
Source: PLoS One. 2024 Jul 25;19(7):e0272239. doi: 10.1371/journal.pone.0272239 (PMC11271938; doi:10.1371/journal.pone.0272239)

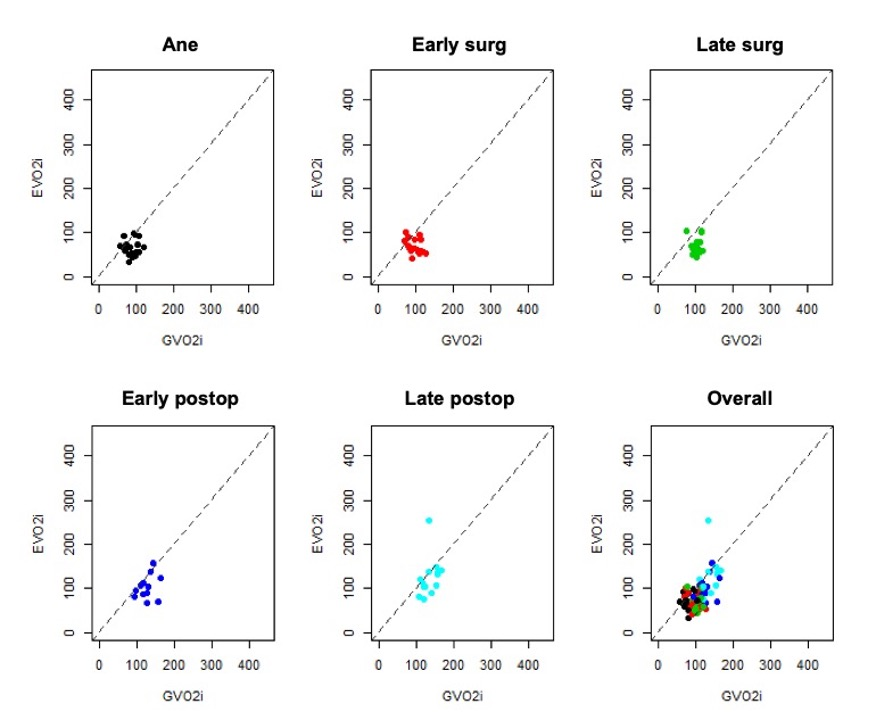

Supplement: S1 File — S1. Two-way single score intraclass correlation, ICC (A,1), of GVO2 and EVO2 indexed for body surface area (i) in ml min-1 m-2 at the different time-points with ICC coefficients (95% CI): a. Anaesthesia (T1; N = 20) -0.066 (-0.187,0.273); b. Early surgery (T2; N = 20) -0.131 (-0.285, 0.212); c. Late surgery (T3; N = 18) 0.019 (-0.081, 0.212); d. Early postop (T4; N = 13) 0.214 (-0.164, 0.619) e. Late postop (T5; N = 14) 0.224 (-0.316, 0.660); f. overall (see text for details). (TIF) [file pone.0272239.s001.tif]
