# Supplementary material for: Perioperative estimations of oxygen consumption from LiDCO™plus-derived cardiac output and Ca-cvO2 difference: Relationship with measurements by indirect calorimetry in elderly patients undergoing major abdominal surgery
Source: PLoS One. 2024 Jul 25;19(7):e0272239. doi: 10.1371/journal.pone.0272239 (PMC11271938; doi:10.1371/journal.pone.0272239)

1. *Mixed effect models for parallellity analysis between GVO2 and EVO2 normalised to baseline Anaesthesia (T0) (= 1.0); Early surgery (T2); Late surgery (T3); Early postop (T4); Late postop (T5) pp.*

The Mixed Procedure

| **Model Information** | |
| --- | --- |
| **Data Set** | WORK.B |
| **Dependent Variable** | Oxygen |
| **Covariance Structure** | Unstructured @ Unstructured |
| **Subject Effect** | ID |
| **Estimation Method** | REML |
| **Residual Variance Method** | None |
| **Fixed Effects SE Method** | Kenward-Roger |
| **Degrees of Freedom Method** | Kenward-Roger |

| **Class Level Information** | | |
| --- | --- | --- |
| **Class** | **Levels** | **Values** |
| **TIME** | 4 | 2 3 4 5 |
| **Meth** | 2 | 1 2 |
| **ID** | 20 | 1 2 3 4 5 6 7 8 9 10 11 12 13 14 15 16 17 18 19 20 |

| **Dimensions** | |
| --- | --- |
| **Covariance Parameters** | 13 |
| **Columns in X** | 15 |
| **Columns in Z** | 0 |
| **Subjects** | 20 |
| **Max Obs per Subject** | 8 |

| **Number of Observations** | |
| --- | --- |
| **Number of Observations Read** | 160 |
| **Number of Observations Used** | 136 |
| **Number of Observations Not Used** | 24 |

| **Iteration History** | | | |
| --- | --- | --- | --- |
| **Iteration** | **Evaluations** | **-2 Res Log Like** | **Criterion** |
| **0** | 1 | 186.59731498 |  |
| **1** | 2 | 178.68264370 | 1.15090650 |
| **2** | 1 | 136.52269380 | 0.37455743 |
| **3** | 1 | 111.77679629 | 0.24917879 |
| **4** | 1 | 91.51244942 | 0.17756900 |
| **5** | 1 | 74.23454847 | 0.12670463 |
| **6** | 1 | 60.92202574 | 0.08340563 |
| **7** | 1 | 51.23189116 | 0.05218360 |
| **8** | 1 | 45.04910304 | 0.02804796 |
| **9** | 1 | 41.60447885 | 0.01225861 |
| **10** | 1 | 40.13613075 | 0.00341512 |
| **11** | 1 | 39.74384373 | 0.00039196 |
| **12** | 1 | 39.70251911 | 0.00000677 |
| **13** | 1 | 39.70184913 | 0.00000000 |

| Convergence criteria met. |
| --- |

| **Covariance Parameter Estimates** | | |
| --- | --- | --- |
| **Cov Parm** | **Subject** | **Estimate** |
| **TIME UN(1,1)** | **ID** | 0.01055 |
| **UN(2,1)** | **ID** | 0.01009 |
| **UN(2,2)** | **ID** | 0.02345 |
| **UN(3,1)** | **ID** | 0.01110 |
| **UN(3,2)** | **ID** | 0.01815 |
| **UN(3,3)** | **ID** | 0.04754 |
| **UN(4,1)** | **ID** | 0.01302 |
| **UN(4,2)** | **ID** | 0.02092 |
| **UN(4,3)** | **ID** | 0.03234 |
| **UN(4,4)** | **ID** | 0.07900 |
| **Meth UN(1,1)** | **ID** | 1.0000 |
| **UN(2,1)** | **ID** | -0.04171 |
| **UN(2,2)** | **ID** | 9.7418 |

| **Fit Statistics** | |
| --- | --- |
| **-2 Res Log Likelihood** | 39.7 |
| **AIC (Smaller is Better)** | 63.7 |
| **AICC (Smaller is Better)** | 66.4 |
| **BIC (Smaller is Better)** | 75.7 |

| **Null Model Likelihood Ratio Test** | | |
| --- | --- | --- |
| **DF** | **Chi-Square** | **Pr > ChiSq** |
| 11 | 146.90 | <.0001 |

| **Solution for Fixed Effects** | | | | | | | |
| --- | --- | --- | --- | --- | --- | --- | --- |
| **Effect** | **TIME** | **Meth** | **Estimate** | **Standard Error** | **DF** | **t Value** | **Pr > \|t\|** |
| **Intercept** |  |  | 2.0739 | 0.2168 | 28.6 | 9.56 | <.0001 |
| **TIME** | **2** |  | -0.9573 | 0.1976 | 27.6 | -4.84 | <.0001 |
| **TIME** | **3** |  | -0.9230 | 0.1940 | 28.9 | -4.76 | <.0001 |
| **TIME** | **4** |  | -0.3628 | 0.2063 | 28.7 | -1.76 | 0.0893 |
| **TIME** | **5** |  | 0 | . | . | . | . |
| **Meth** |  | **1** | -0.5141 | 0.2282 | 25.4 | -2.25 | 0.0331 |
| **Meth** |  | **2** | 0 | . | . | . | . |
| **TIME*Meth** | **2** | **1** | 0.4971 | 0.2082 | 24.7 | 2.39 | 0.0249 |
| **TIME*Meth** | **2** | **2** | 0 | . | . | . | . |
| **TIME*Meth** | **3** | **1** | 0.5559 | 0.2044 | 25.1 | 2.72 | 0.0117 |
| **TIME*Meth** | **3** | **2** | 0 | . | . | . | . |
| **TIME*Meth** | **4** | **1** | 0.2642 | 0.2187 | 27.6 | 1.21 | 0.2373 |
| **TIME*Meth** | **4** | **2** | 0 | . | . | . | . |
| **TIME*Meth** | **5** | **1** | 0 | . | . | . | . |
| **TIME*Meth** | **5** | **2** | 0 | . | . | . | . |

| **Solution for Fixed Effects** | | | | | | | |
| --- | --- | --- | --- | --- | --- | --- | --- |
| **Effect** | **TIME** | **Meth** | **Estimate** | **Standard Error** | **DF** | **t Value** | **Pr > \|t\|** |
| **Intercept** |  |  | 1.1166 | 0.07364 | 29.2 | 15.16 | <.0001 |
| **TIME** | **3** |  | 0.03426 | 0.08756 | 16.3 | 0.39 | 0.7006 |
| **TIME** | **4** |  | 0.5944 | 0.1554 | 16.8 | 3.83 | 0.0014 |
| **TIME** | **5** |  | 0.9573 | 0.1976 | 27.6 | 4.84 | <.0001 |
| **TIME** | **2** |  | 0 | . | . | . | . |
| **Meth** |  | **1** | -0.01698 | 0.07721 | 27.1 | -0.22 | 0.8276 |
| **Meth** |  | **2** | 0 | . | . | . | . |
| **TIME*Meth** | **3** | **1** | 0.05884 | 0.09249 | 20.6 | 0.64 | 0.5317 |
| **TIME*Meth** | **3** | **2** | 0 | . | . | . | . |
| **TIME*Meth** | **4** | **1** | -0.2329 | 0.1647 | 18.6 | -1.41 | 0.1738 |
| **TIME*Meth** | **4** | **2** | 0 | . | . | . | . |
| **TIME*Meth** | **5** | **1** | -0.4971 | 0.2082 | 24.7 | -2.39 | 0.0249 |
| **TIME*Meth** | **5** | **2** | 0 | . | . | . | . |
| **TIME*Meth** | **2** | **1** | 0 | . | . | . | . |
| **TIME*Meth** | **2** | **2** | 0 | . | . | . | . |

| **Type 3 Tests of Fixed Effects** | | | | |
| --- | --- | --- | --- | --- |
| **Effect** | **Num DF** | **Den DF** | **F Value** | **Pr > F** |
| **TIME** | 3 | 18.1 | 19.30 | <.0001 |
| **Meth** | 1 | 24.1 | 2.41 | 0.1338 |
| **TIME*Meth** | 3 | 20.4 | 2.60 | 0.0802 |

| **Least Squares Means** | | | | | | | | | | |
| --- | --- | --- | --- | --- | --- | --- | --- | --- | --- | --- |
| **Effect** | **TIME** | **Meth** | **Estimate** | **Standard Error** | **DF** | **t Value** | **Pr > \|t\|** | **Alpha** | **Lower** | **Upper** |
| **TIME*Meth** | **2** | **1** | 1.0996 | 0.02296 | 22.6 | 47.89 | <.0001 | 0.05 | 1.0521 | 1.1472 |
| **TIME*Meth** | **2** | **2** | 1.1166 | 0.07364 | 29.2 | 15.16 | <.0001 | 0.05 | 0.9660 | 1.2672 |
| **TIME*Meth** | **3** | **1** | 1.1927 | 0.03540 | 29.3 | 33.69 | <.0001 | 0.05 | 1.1204 | 1.2651 |
| **TIME*Meth** | **3** | **2** | 1.1509 | 0.1124 | 22.5 | 10.24 | <.0001 | 0.05 | 0.9180 | 1.3838 |
| **TIME*Meth** | **4** | **1** | 1.4612 | 0.05809 | 27.8 | 25.15 | <.0001 | 0.05 | 1.3421 | 1.5802 |
| **TIME*Meth** | **4** | **2** | 1.7111 | 0.1728 | 17.7 | 9.90 | <.0001 | 0.05 | 1.3475 | 2.0746 |
| **TIME*Meth** | **5** | **1** | 1.5598 | 0.07097 | 18 | 21.98 | <.0001 | 0.05 | 1.4107 | 1.7089 |
| **TIME*Meth** | **5** | **2** | 2.0739 | 0.2168 | 28.6 | 9.56 | <.0001 | 0.05 | 1.6302 | 2.5176 |


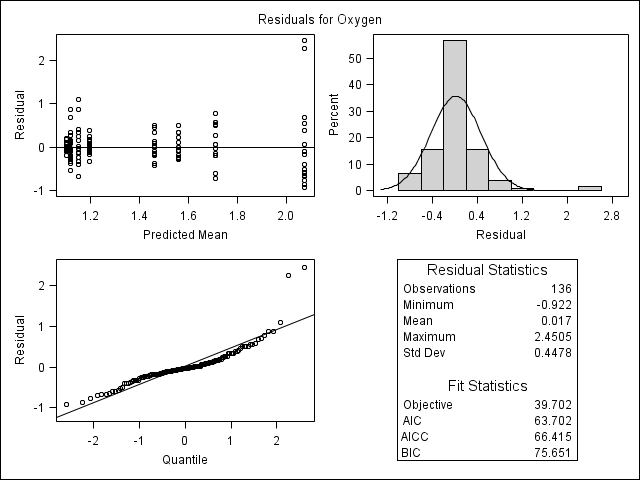


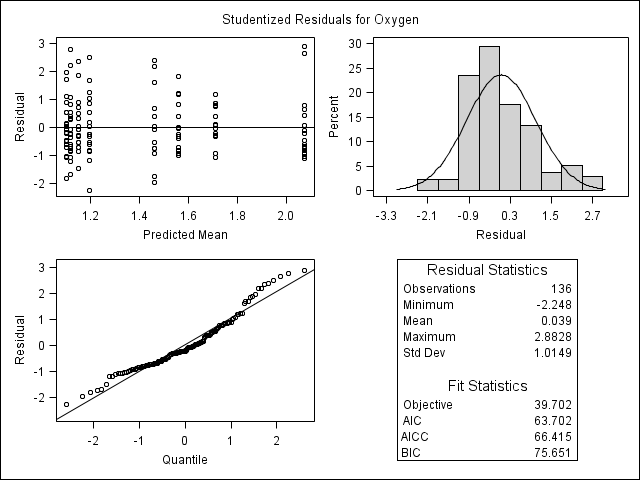


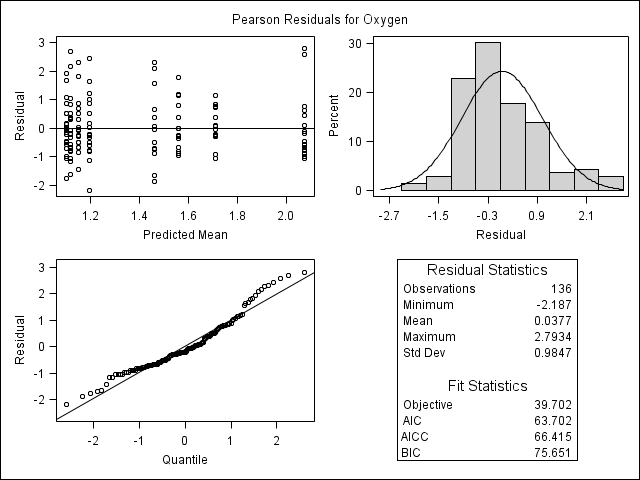


1. *Mixed effect models for parallellity analysis between GVO2 and Ca-cvO2 (arterio-central venous oxygen content difference) normalised to baseline Anaesthesia (T0) (= 1.0); Early surgery (T2); Late surgery (T3); Early postop (T4); Late postop (T5)pp.*

The Mixed Procedure

| **Model Information** | |
| --- | --- |
| **Data Set** | WORK.B |
| **Dependent Variable** | Oxygen |
| **Covariance Structure** | Unstructured @ Unstructured |
| **Subject Effect** | ID |
| **Estimation Method** | REML |
| **Residual Variance Method** | None |
| **Fixed Effects SE Method** | Kenward-Roger |
| **Degrees of Freedom Method** | Kenward-Roger |

| **Class Level Information** | | |
| --- | --- | --- |
| **Class** | **Levels** | **Values** |
| **TIME** | 4 | 2 3 4 5 |
| **Meth** | 2 | 1 2 |
| **ID** | 20 | 1 2 3 4 5 6 7 8 9 10 11 12 13 14 15 16 17 18 19 20 |

| **Dimensions** | |
| --- | --- |
| **Covariance Parameters** | 13 |
| **Columns in X** | 15 |
| **Columns in Z** | 0 |
| **Subjects** | 20 |
| **Max Obs per Subject** | 8 |

| **Number of Observations** | |
| --- | --- |
| **Number of Observations Read** | 160 |
| **Number of Observations Used** | 136 |
| **Number of Observations Not Used** | 24 |

| **Iteration History** | | | |
| --- | --- | --- | --- |
| **Iteration** | **Evaluations** | **-2 Res Log Like** | **Criterion** |
| **0** | 1 | 46.98313789 |  |
| **1** | 2 | 400.30607782 | 1.78399731 |
| **2** | 1 | 209.45161829 | 5.54655794 |
| **3** | 1 | 117.21525887 | 0.57405760 |
| **4** | 1 | 73.12467483 | 0.24634276 |
| **5** | 1 | 46.57770025 | 0.17763882 |
| **6** | 1 | 24.20090260 | 0.13694511 |
| **7** | 1 | 4.67351059 | 0.10249963 |
| **8** | 1 | -10.96082637 | 0.06986345 |
| **9** | 1 | -22.37774847 | 0.04308018 |
| **10** | 1 | -29.56950623 | 0.02225387 |
| **11** | 1 | -33.34171422 | 0.00898353 |
| **12** | 1 | -34.82839070 | 0.00223120 |
| **13** | 1 | -35.17844269 | 0.00020591 |
| **14** | 1 | -35.20808108 | 0.00000229 |
| **15** | 1 | -35.20839311 | 0.00000000 |

| Convergence criteria met. |
| --- |

| **Covariance Parameter Estimates** | | |
| --- | --- | --- |
| **Cov Parm** | **Subject** | **Estimate** |
| **TIME UN(1,1)** | **ID** | 0.01183 |
| **UN(2,1)** | **ID** | 0.009075 |
| **UN(2,2)** | **ID** | 0.02118 |
| **UN(3,1)** | **ID** | 0.01335 |
| **UN(3,2)** | **ID** | 0.01636 |
| **UN(3,3)** | **ID** | 0.05072 |
| **UN(4,1)** | **ID** | 0.009617 |
| **UN(4,2)** | **ID** | 0.02187 |
| **UN(4,3)** | **ID** | 0.02212 |
| **UN(4,4)** | **ID** | 0.05707 |
| **Meth UN(1,1)** | **ID** | 1.0000 |
| **UN(2,1)** | **ID** | -0.6643 |
| **UN(2,2)** | **ID** | 3.8915 |

| **Fit Statistics** | |
| --- | --- |
| **-2 Res Log Likelihood** | -35.2 |
| **AIC (Smaller is Better)** | -11.2 |
| **AICC (Smaller is Better)** | -8.5 |
| **BIC (Smaller is Better)** | 0.7 |

| **Null Model Likelihood Ratio Test** | | |
| --- | --- | --- |
| **DF** | **Chi-Square** | **Pr > ChiSq** |
| 11 | 82.19 | <.0001 |

| **Solution for Fixed Effects** | | | | | | | |
| --- | --- | --- | --- | --- | --- | --- | --- |
| **Effect** | **TIME** | **Meth** | **Estimate** | **Standard Error** | **DF** | **t Value** | **Pr > \|t\|** |
| **Intercept** |  |  | 1.1615 | 0.1149 | 22.3 | 10.11 | <.0001 |
| **TIME** | **2** |  | -0.2091 | 0.1085 | 24 | -1.93 | 0.0659 |
| **TIME** | **3** |  | -0.1753 | 0.09143 | 23.4 | -1.92 | 0.0675 |
| **TIME** | **4** |  | 0.1009 | 0.1302 | 28 | 0.78 | 0.4447 |
| **TIME** | **5** |  | 0 | . | . | . | . |
| **Meth** |  | **1** | 0.3979 | 0.1441 | 20.2 | 2.76 | 0.0120 |
| **Meth** |  | **2** | 0 | . | . | . | . |
| **TIME*Meth** | **2** | **1** | -0.2507 | 0.1361 | 21.4 | -1.84 | 0.0794 |
| **TIME*Meth** | **2** | **2** | 0 | . | . | . | . |
| **TIME*Meth** | **3** | **1** | -0.1900 | 0.1148 | 20.6 | -1.66 | 0.1130 |
| **TIME*Meth** | **3** | **2** | 0 | . | . | . | . |
| **TIME*Meth** | **4** | **1** | -0.1914 | 0.1646 | 24.7 | -1.16 | 0.2561 |
| **TIME*Meth** | **4** | **2** | 0 | . | . | . | . |
| **TIME*Meth** | **5** | **1** | 0 | . | . | . | . |
| **TIME*Meth** | **5** | **2** | 0 | . | . | . | . |

| **Solution for Fixed Effects** | | | | | | | |
| --- | --- | --- | --- | --- | --- | --- | --- |
| **Effect** | **TIME** | **Meth** | **Estimate** | **Standard Error** | **DF** | **t Value** | **Pr > \|t\|** |
| **Intercept** |  |  | 0.9524 | 0.04910 | 30.1 | 19.40 | <.0001 |
| **TIME** | **3** |  | 0.03380 | 0.05746 | 22.7 | 0.59 | 0.5621 |
| **TIME** | **4** |  | 0.3100 | 0.09921 | 19.7 | 3.12 | 0.0054 |
| **TIME** | **5** |  | 0.2091 | 0.1085 | 24 | 1.93 | 0.0659 |
| **TIME** | **2** |  | 0 | . | . | . | . |
| **Meth** |  | **1** | 0.1472 | 0.06150 | 26.9 | 2.39 | 0.0239 |
| **Meth** |  | **2** | 0 | . | . | . | . |
| **TIME*Meth** | **3** | **1** | 0.06067 | 0.07242 | 28.3 | 0.84 | 0.4091 |
| **TIME*Meth** | **3** | **2** | 0 | . | . | . | . |
| **TIME*Meth** | **4** | **1** | 0.05933 | 0.1264 | 22.4 | 0.47 | 0.6432 |
| **TIME*Meth** | **4** | **2** | 0 | . | . | . | . |
| **TIME*Meth** | **5** | **1** | 0.2507 | 0.1361 | 21.4 | 1.84 | 0.0794 |
| **TIME*Meth** | **5** | **2** | 0 | . | . | . | . |
| **TIME*Meth** | **2** | **1** | 0 | . | . | . | . |
| **TIME*Meth** | **2** | **2** | 0 | . | . | . | . |

| **Type 3 Tests of Fixed Effects** | | | | |
| --- | --- | --- | --- | --- |
| **Effect** | **Num DF** | **Den DF** | **F Value** | **Pr > F** |
| **TIME** | 3 | 19.9 | 23.91 | <.0001 |
| **Meth** | 1 | 24 | 8.18 | 0.0086 |
| **TIME*Meth** | 3 | 21.9 | 1.06 | 0.3849 |

| **Least Squares Means** | | | | | | | | | | |
| --- | --- | --- | --- | --- | --- | --- | --- | --- | --- | --- |
| **Effect** | **TIME** | **Meth** | **Estimate** | **Standard Error** | **DF** | **t Value** | **Pr > \|t\|** | **Alpha** | **Lower** | **Upper** |
| **TIME*Meth** | **2** | **1** | 1.0996 | 0.02432 | 22.8 | 45.21 | <.0001 | 0.05 | 1.0493 | 1.1500 |
| **TIME*Meth** | **2** | **2** | 0.9524 | 0.04910 | 30.1 | 19.40 | <.0001 | 0.05 | 0.8522 | 1.0527 |
| **TIME*Meth** | **3** | **1** | 1.1941 | 0.03380 | 30.8 | 35.32 | <.0001 | 0.05 | 1.1251 | 1.2631 |
| **TIME*Meth** | **3** | **2** | 0.9862 | 0.06756 | 23.7 | 14.60 | <.0001 | 0.05 | 0.8467 | 1.1258 |
| **TIME*Meth** | **4** | **1** | 1.4689 | 0.05986 | 27.2 | 24.54 | <.0001 | 0.05 | 1.3462 | 1.5917 |
| **TIME*Meth** | **4** | **2** | 1.2624 | 0.1131 | 21.4 | 11.17 | <.0001 | 0.05 | 1.0275 | 1.4973 |
| **TIME*Meth** | **5** | **1** | 1.5594 | 0.05969 | 21.9 | 26.13 | <.0001 | 0.05 | 1.4356 | 1.6832 |
| **TIME*Meth** | **5** | **2** | 1.1615 | 0.1149 | 22.3 | 10.11 | <.0001 | 0.05 | 0.9234 | 1.3996 |


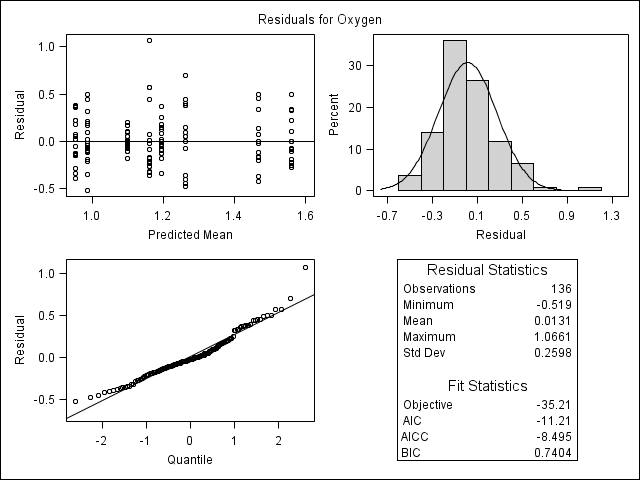


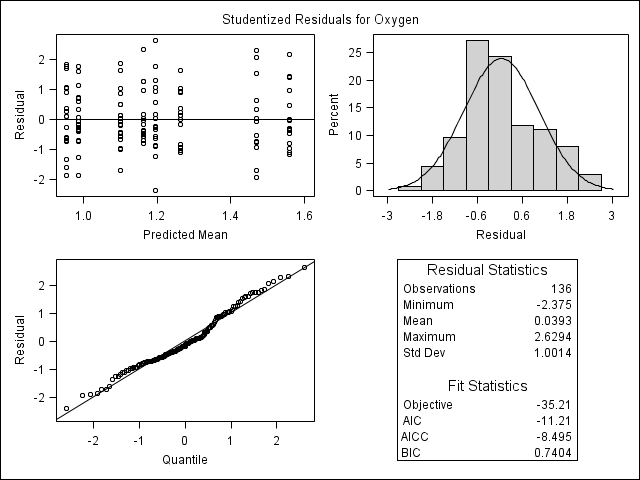


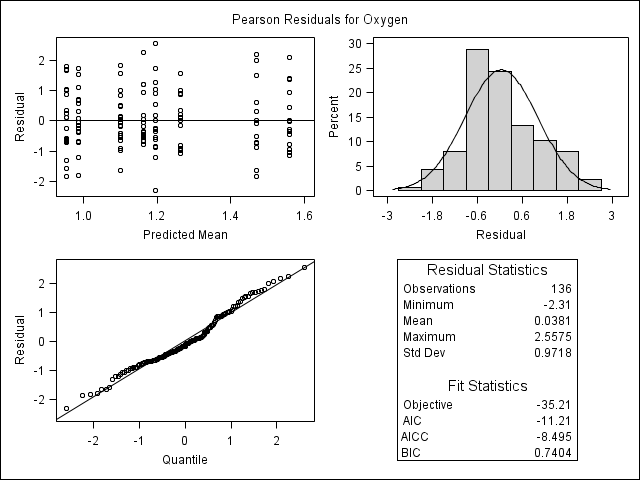


1. *Mixed effect models for parallellity analysis between GVO2 and CI (cardiac index) normalised to baseline Anaesthesia (T0) (= 1.0); Early surgery (T2); Late surgery (T3); Early postop (T4); Late postop (T5) pp.*

The Mixed Procedure

| **Model Information** | |
| --- | --- |
| **Data Set** | WORK.B |
| **Dependent Variable** | Oxygen |
| **Covariance Structure** | Unstructured @ Unstructured |
| **Subject Effect** | ID |
| **Estimation Method** | REML |
| **Residual Variance Method** | None |
| **Fixed Effects SE Method** | Kenward-Roger |
| **Degrees of Freedom Method** | Kenward-Roger |

| **Class Level Information** | | |
| --- | --- | --- |
| **Class** | **Levels** | **Values** |
| **TIME** | 4 | 2 3 4 5 |
| **Meth** | 2 | 1 2 |
| **ID** | 20 | 1 2 3 4 5 6 7 8 9 10 11 12 13 14 15 16 17 18 19 20 |

| **Dimensions** | |
| --- | --- |
| **Covariance Parameters** | 13 |
| **Columns in X** | 15 |
| **Columns in Z** | 0 |
| **Subjects** | 20 |
| **Max Obs per Subject** | 8 |

| **Number of Observations** | |
| --- | --- |
| **Number of Observations Read** | 160 |
| **Number of Observations Used** | 136 |
| **Number of Observations Not Used** | 24 |

| **Iteration History** | | | |
| --- | --- | --- | --- |
| **Iteration** | **Evaluations** | **-2 Res Log Like** | **Criterion** |
| **0** | 1 | 95.15204603 |  |
| **1** | 2 | 331.55159403 | 2.66963135 |
| **2** | 1 | 165.24316350 | 1.73802321 |
| **3** | 1 | 87.12608959 | 0.37206349 |
| **4** | 1 | 51.32253227 | 0.18374640 |
| **5** | 1 | 28.94369627 | 0.13664353 |
| **6** | 1 | 10.04470707 | 0.10213311 |
| **7** | 1 | -5.31786552 | 0.07218866 |
| **8** | 1 | -16.83140645 | 0.04618334 |
| **9** | 1 | -24.44264948 | 0.02483430 |
| **10** | 1 | -28.56345850 | 0.00984933 |
| **11** | 1 | -30.15775880 | 0.00221114 |
| **12** | 1 | -30.49384210 | 0.00015690 |
| **13** | 1 | -30.51572535 | 0.00000099 |
| **14** | 1 | -30.51585775 | 0.00000000 |

| Convergence criteria met. |
| --- |

| **Covariance Parameter Estimates** | | |
| --- | --- | --- |
| **Cov Parm** | **Subject** | **Estimate** |
| **TIME UN(1,1)** | **ID** | 0.008156 |
| **UN(2,1)** | **ID** | 0.006363 |
| **UN(2,2)** | **ID** | 0.02104 |
| **UN(3,1)** | **ID** | 0.009276 |
| **UN(3,2)** | **ID** | 0.01921 |
| **UN(3,3)** | **ID** | 0.06686 |
| **UN(4,1)** | **ID** | 0.005426 |
| **UN(4,2)** | **ID** | 0.01695 |
| **UN(4,3)** | **ID** | 0.04631 |
| **UN(4,4)** | **ID** | 0.05014 |
| **Meth UN(1,1)** | **ID** | 1.0000 |
| **UN(2,1)** | **ID** | 0.7234 |
| **UN(2,2)** | **ID** | 5.2357 |

| **Fit Statistics** | |
| --- | --- |
| **-2 Res Log Likelihood** | -30.5 |
| **AIC (Smaller is Better)** | -6.5 |
| **AICC (Smaller is Better)** | -3.8 |
| **BIC (Smaller is Better)** | 5.4 |

| **Null Model Likelihood Ratio Test** | | |
| --- | --- | --- |
| **DF** | **Chi-Square** | **Pr > ChiSq** |
| 11 | 125.67 | <.0001 |

| **Solution for Fixed Effects** | | | | | | | |
| --- | --- | --- | --- | --- | --- | --- | --- |
| **Effect** | **TIME** | **Meth** | **Estimate** | **Standard Error** | **DF** | **t Value** | **Pr > \|t\|** |
| **Intercept** |  |  | 1.7978 | 0.1242 | 23 | 14.47 | <.0001 |
| **TIME** | **2** |  | -0.6203 | 0.1217 | 23.4 | -5.10 | <.0001 |
| **TIME** | **3** |  | -0.6359 | 0.1086 | 25.9 | -5.85 | <.0001 |
| **TIME** | **4** |  | -0.3317 | 0.09745 | 18.6 | -3.40 | 0.0031 |
| **TIME** | **5** |  | 0 | . | . | . | . |
| **Meth** |  | **1** | -0.2291 | 0.1201 | 24.3 | -1.91 | 0.0683 |
| **Meth** |  | **2** | 0 | . | . | . | . |
| **TIME*Meth** | **2** | **1** | 0.1513 | 0.1176 | 24.5 | 1.29 | 0.2105 |
| **TIME*Meth** | **2** | **2** | 0 | . | . | . | . |
| **TIME*Meth** | **3** | **1** | 0.2613 | 0.1052 | 26.1 | 2.48 | 0.0198 |
| **TIME*Meth** | **3** | **2** | 0 | . | . | . | . |
| **TIME*Meth** | **4** | **1** | 0.2205 | 0.09662 | 20.7 | 2.28 | 0.0332 |
| **TIME*Meth** | **4** | **2** | 0 | . | . | . | . |
| **TIME*Meth** | **5** | **1** | 0 | . | . | . | . |
| **TIME*Meth** | **5** | **2** | 0 | . | . | . | . |

| **Solution for Fixed Effects** | | | | | | | |
| --- | --- | --- | --- | --- | --- | --- | --- |
| **Effect** | **TIME** | **Meth** | **Estimate** | **Standard Error** | **DF** | **t Value** | **Pr > \|t\|** |
| **Intercept** |  |  | 1.1775 | 0.04710 | 30.1 | 25.00 | <.0001 |
| **TIME** | **3** |  | -0.01561 | 0.06990 | 18.1 | -0.22 | 0.8258 |
| **TIME** | **4** |  | 0.2886 | 0.1373 | 25.2 | 2.10 | 0.0457 |
| **TIME** | **5** |  | 0.6203 | 0.1217 | 23.4 | 5.10 | <.0001 |
| **TIME** | **2** |  | 0 | . | . | . | . |
| **Meth** |  | **1** | -0.07787 | 0.04487 | 23.2 | -1.74 | 0.0959 |
| **Meth** |  | **2** | 0 | . | . | . | . |
| **TIME*Meth** | **3** | **1** | 0.1100 | 0.06726 | 22.7 | 1.64 | 0.1158 |
| **TIME*Meth** | **3** | **2** | 0 | . | . | . | . |
| **TIME*Meth** | **4** | **1** | 0.06927 | 0.1324 | 25.2 | 0.52 | 0.6054 |
| **TIME*Meth** | **4** | **2** | 0 | . | . | . | . |
| **TIME*Meth** | **5** | **1** | -0.1513 | 0.1176 | 24.5 | -1.29 | 0.2105 |
| **TIME*Meth** | **5** | **2** | 0 | . | . | . | . |
| **TIME*Meth** | **2** | **1** | 0 | . | . | . | . |
| **TIME*Meth** | **2** | **2** | 0 | . | . | . | . |

| **Type 3 Tests of Fixed Effects** | | | | |
| --- | --- | --- | --- | --- |
| **Effect** | **Num DF** | **Den DF** | **F Value** | **Pr > F** |
| **TIME** | 3 | 18.1 | 20.50 | <.0001 |
| **Meth** | 1 | 24.7 | 0.83 | 0.3702 |
| **TIME*Meth** | 3 | 21.4 | 3.52 | 0.0326 |

| **Least Squares Means** | | | | | | | | | | |
| --- | --- | --- | --- | --- | --- | --- | --- | --- | --- | --- |
| **Effect** | **TIME** | **Meth** | **Estimate** | **Standard Error** | **DF** | **t Value** | **Pr > \|t\|** | **Alpha** | **1.** | **Upper** |
| **TIME*Meth** | **2** | **1** | 1.0996 | 0.02019 | 26.2 | 54.45 | <.0001 | 0.05 | 1.0581 | 1.1411 |
| **TIME*Meth** | **2** | **2** | 1.1775 | 0.04710 | 30.1 | 25.00 | <.0001 | 0.05 | 1.0813 | 1.2737 |
| **TIME*Meth** | **3** | **1** | 1.1940 | 0.03386 | 30.3 | 35.27 | <.0001 | 0.05 | 1.1249 | 1.2631 |
| **TIME*Meth** | **3** | **2** | 1.1619 | 0.07817 | 20.4 | 14.86 | <.0001 | 0.05 | 0.9990 | 1.3247 |
| **TIME*Meth** | **4** | **1** | 1.4575 | 0.06536 | 25.7 | 22.30 | <.0001 | 0.05 | 1.3230 | 1.5919 |
| **TIME*Meth** | **4** | **2** | 1.4661 | 0.1470 | 26.6 | 9.98 | <.0001 | 0.05 | 1.1644 | 1.7678 |
| **TIME*Meth** | **5** | **1** | 1.5687 | 0.05488 | 23.5 | 28.59 | <.0001 | 0.05 | 1.4553 | 1.6821 |
| **TIME*Meth** | **5** | **2** | 1.7978 | 0.1242 | 23 | 14.47 | <.0001 | 0.05 | 1.5408 | 2.0548 |


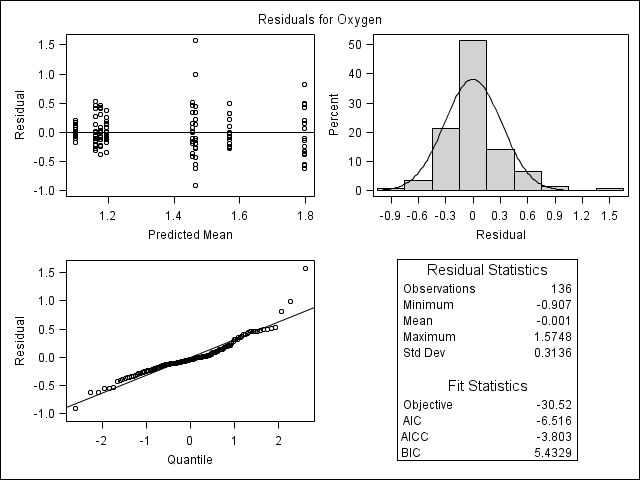


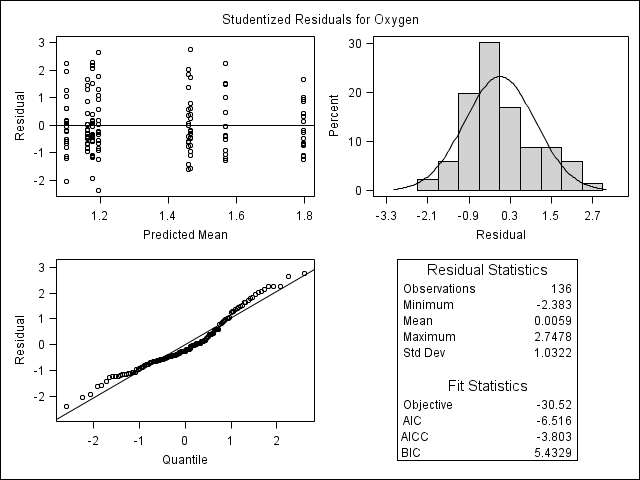


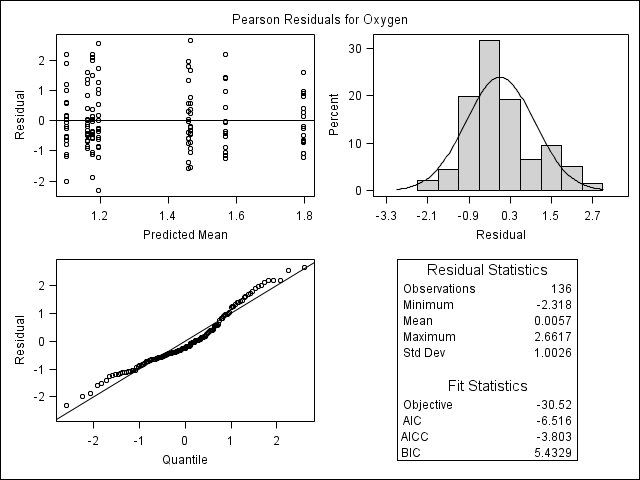


1. *Mixed effect models for parallellity analysis between GVO2 and EVO2 normalised to overall mean for each method (= 1.0) in anaesthetized subjects Anaesthesia (T0); Early surgery (T2); Late surgery (T3) pp.*

The Mixed Procedure

| **Model Information** | |
| --- | --- |
| **Data Set** | WORK.B |
| **Dependent Variable** | Oxygen |
| **Covariance Structure** | Unstructured @ Autoregressive |
| **Subject Effect** | ID |
| **Estimation Method** | REML |
| **Residual Variance Method** | None |
| **Fixed Effects SE Method** | Kenward-Roger |
| **Degrees of Freedom Method** | Kenward-Roger |

| **Class Level Information** | | |
| --- | --- | --- |
| **Class** | **Levels** | **Values** |
| **OPTYP** | 3 | L P X |
| **TIME** | 3 | 2 3 1 |
| **Meth** | 2 | 1 2 |
| **ID** | 20 | 1 2 3 4 5 6 7 8 9 10 11 12 13 14 15 16 17 18 19 20 |

| **Dimensions** | |
| --- | --- |
| **Covariance Parameters** | 4 |
| **Columns in X** | 12 |
| **Columns in Z** | 0 |
| **Subjects** | 20 |
| **Max Obs per Subject** | 6 |

| **Number of Observations** | |
| --- | --- |
| **Number of Observations Read** | 120 |
| **Number of Observations Used** | 116 |
| **Number of Observations Not Used** | 4 |

| **Iteration History** | | | |
| --- | --- | --- | --- |
| **Iteration** | **Evaluations** | **-2 Res Log Like** | **Criterion** |
| **0** | 1 | -7.28459988 |  |
| **1** | 4 | -48.22626425 | 0.00452813 |
| **2** | 1 | -48.87486996 | 0.00042014 |
| **3** | 1 | -48.93138180 | 0.00000589 |
| **4** | 1 | -48.93212826 | 0.00000000 |

| Convergence criteria met. |
| --- |

| **Covariance Parameter Estimates** | | |
| --- | --- | --- |
| **Cov Parm** | **Subject** | **Estimate** |
| **Meth UN(1,1)** | **ID** | 0.02077 |
| **UN(2,1)** | **ID** | -0.01280 |
| **UN(2,2)** | **ID** | 0.09315 |
| **TIME AR(1)** | **ID** | 0.5959 |

| **Fit Statistics** | |
| --- | --- |
| **-2 Res Log Likelihood** | -48.9 |
| **AIC (Smaller is Better)** | -40.9 |
| **AICC (Smaller is Better)** | -40.6 |
| **BIC (Smaller is Better)** | -36.9 |

| **Null Model Likelihood Ratio Test** | | |
| --- | --- | --- |
| **DF** | **Chi-Square** | **Pr > ChiSq** |
| 3 | 41.65 | <.0001 |

| **Solution for Fixed Effects** | | | | | | | |
| --- | --- | --- | --- | --- | --- | --- | --- |
| **Effect** | **TIME** | **Meth** | **Estimate** | **Standard Error** | **DF** | **t Value** | **Pr > \|t\|** |
| **Intercept** |  |  | 0.9624 | 0.06824 | 32.3 | 14.10 | <.0001 |
| **TIME** | **2** |  | 0.05231 | 0.07902 | 54.9 | 0.66 | 0.5107 |
| **TIME** | **3** |  | 0.07868 | 0.06447 | 49.3 | 1.22 | 0.2281 |
| **TIME** | **1** |  | 0 | . | . | . | . |
| **Meth** |  | **1** | -0.04187 | 0.08352 | 31.9 | -0.50 | 0.6196 |
| **Meth** |  | **2** | 0 | . | . | . | . |
| **TIME*Meth** | **2** | **1** | 0.03303 | 0.09672 | 55 | 0.34 | 0.7340 |
| **TIME*Meth** | **2** | **2** | 0 | . | . | . | . |
| **TIME*Meth** | **3** | **1** | 0.07989 | 0.07892 | 49.6 | 1.01 | 0.3163 |
| **TIME*Meth** | **3** | **2** | 0 | . | . | . | . |
| **TIME*Meth** | **1** | **1** | 0 | . | . | . | . |
| **TIME*Meth** | **1** | **2** | 0 | . | . | . | . |

| **Type 3 Tests of Fixed Effects** | | | | |
| --- | --- | --- | --- | --- |
| **Effect** | **Num DF** | **Den DF** | **F Value** | **Pr > F** |
| **TIME** | 2 | 42.6 | 7.43 | 0.0017 |
| **Meth** | 1 | 19.8 | 0.00 | 0.9513 |
| **TIME*Meth** | 2 | 49.9 | 0.57 | 0.5669 |

| **Least Squares Means** | | | | | | | | | | |
| --- | --- | --- | --- | --- | --- | --- | --- | --- | --- | --- |
| **Effect** | **TIME** | **Meth** | **Estimate** | **Standard Error** | **DF** | **t Value** | **Pr > \|t\|** | **Alpha** | **Lower** | **Upper** |
| **TIME*Meth** | **2** | **1** | 1.0059 | 0.03222 | 47.5 | 31.22 | <.0001 | 0.05 | 0.9411 | 1.0707 |
| **TIME*Meth** | **2** | **2** | 1.0147 | 0.06824 | 32.3 | 14.87 | <.0001 | 0.05 | 0.8757 | 1.1537 |
| **TIME*Meth** | **3** | **1** | 1.0791 | 0.03308 | 49.5 | 32.62 | <.0001 | 0.05 | 1.0126 | 1.1456 |
| **TIME*Meth** | **3** | **2** | 1.0411 | 0.07010 | 34.4 | 14.85 | <.0001 | 0.05 | 0.8987 | 1.1835 |
| **TIME*Meth** | **1** | **1** | 0.9205 | 0.03222 | 47.5 | 28.57 | <.0001 | 0.05 | 0.8557 | 0.9853 |
| **TIME*Meth** | **1** | **2** | 0.9624 | 0.06824 | 32.3 | 14.10 | <.0001 | 0.05 | 0.8234 | 1.1013 |


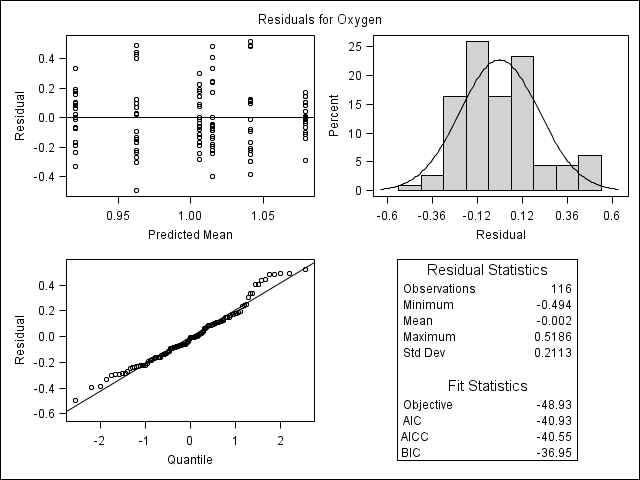


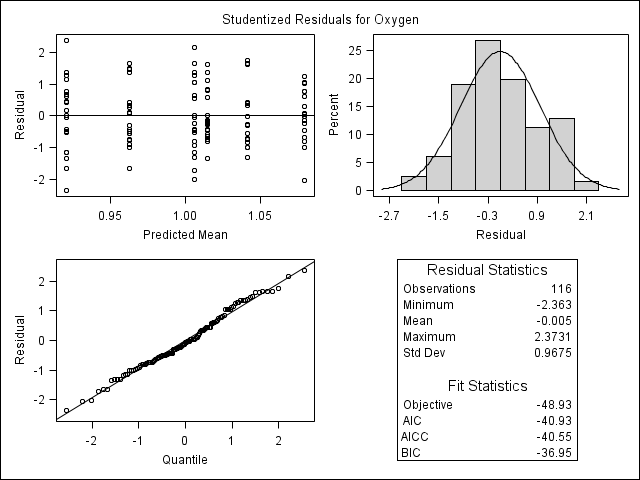


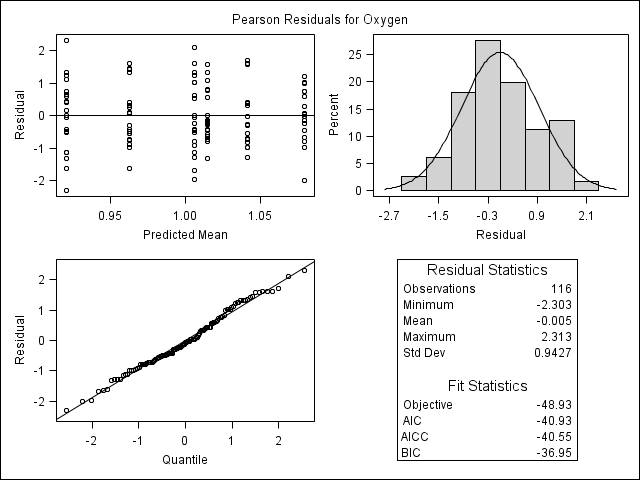


1. *Mixed effect models for parallellity analysis between GVO2 and EVO2 normalised to overall mean for each method (= 1.0) in awake subjects Early postop (T4); Late postop (T5)pp.*

The Mixed Procedure

| **Model Information** | |
| --- | --- |
| **Data Set** | WORK.C |
| **Dependent Variable** | Oxygen |
| **Covariance Structure** | Unstructured @ Unstructured |
| **Subject Effect** | ID |
| **Estimation Method** | REML |
| **Residual Variance Method** | None |
| **Fixed Effects SE Method** | Kenward-Roger |
| **Degrees of Freedom Method** | Kenward-Roger |

| **Class Level Information** | | |
| --- | --- | --- |
| **Class** | **Levels** | **Values** |
| **OPTYP** | 3 | L P X |
| **TIME** | 2 | 5 4 |
| **Meth** | 2 | 1 2 |
| **ID** | 20 | 1 2 3 4 5 6 7 8 9 10 11 12 13 14 15 16 17 18 19 20 |

| **Dimensions** | |
| --- | --- |
| **Covariance Parameters** | 6 |
| **Columns in X** | 9 |
| **Columns in Z** | 0 |
| **Subjects** | 20 |
| **Max Obs per Subject** | 4 |

| **Number of Observations** | |
| --- | --- |
| **Number of Observations Read** | 79 |
| **Number of Observations Used** | 59 |
| **Number of Observations Not Used** | 20 |

| **Iteration History** | | | |
| --- | --- | --- | --- |
| **Iteration** | **Evaluations** | **-2 Res Log Like** | **Criterion** |
| **0** | 1 | -8.21371710 |  |
| **1** | 2 | 278.82460592 | 1.28602876 |
| **2** | 1 | 129.93812050 | 4.06054959 |
| **3** | 1 | 54.03890990 | 1.19928035 |
| **4** | 1 | 17.73418068 | 0.31258848 |
| **5** | 1 | 0.83042319 | 0.15144805 |
| **6** | 1 | -9.38459324 | 0.10624097 |
| **7** | 1 | -17.02867400 | 0.06489433 |
| **8** | 1 | -22.05385368 | 0.03363969 |
| **9** | 1 | -24.68186176 | 0.01297665 |
| **10** | 1 | -25.68836424 | 0.00314403 |
| **11** | 1 | -25.91937092 | 0.00028443 |
| **12** | 1 | -25.93859792 | 0.00000317 |
| **13** | 1 | -25.93880058 | 0.00000000 |

| Convergence criteria met. |
| --- |

| **Covariance Parameter Estimates** | | |
| --- | --- | --- |
| **Cov Parm** | **Subject** | **Estimate** |
| **Meth UN(1,1)** | **ID** | 0.01902 |
| **UN(2,1)** | **ID** | 0.01446 |
| **UN(2,2)** | **ID** | 0.05971 |
| **TIME UN(1,1)** | **ID** | 1.0000 |
| **UN(2,1)** | **ID** | 0.5697 |
| **UN(2,2)** | **ID** | 1.1594 |

| **Fit Statistics** | |
| --- | --- |
| **-2 Res Log Likelihood** | -25.9 |
| **AIC (Smaller is Better)** | -15.9 |
| **AICC (Smaller is Better)** | -14.7 |
| **BIC (Smaller is Better)** | -11.0 |

| **Null Model Likelihood Ratio Test** | | |
| --- | --- | --- |
| **DF** | **Chi-Square** | **Pr > ChiSq** |
| 4 | 17.73 | 0.0014 |

| **Solution for Fixed Effects** | | | | | | | |
| --- | --- | --- | --- | --- | --- | --- | --- |
| **Effect** | **TIME** | **Meth** | **Estimate** | **Standard Error** | **DF** | **t Value** | **Pr > \|t\|** |
| **Intercept** |  |  | 0.9247 | 0.06854 | 16.6 | 13.49 | <.0001 |
| **TIME** | **5** |  | 0.08108 | 0.06856 | 20.9 | 1.18 | 0.2503 |
| **TIME** | **4** |  | 0 | . | . | . | . |
| **Meth** |  | **1** | 0.03525 | 0.06456 | 18.7 | 0.55 | 0.5916 |
| **Meth** |  | **2** | 0 | . | . | . | . |
| **TIME*Meth** | **5** | **1** | -0.00334 | 0.06655 | 22 | -0.05 | 0.9604 |
| **TIME*Meth** | **5** | **2** | 0 | . | . | . | . |
| **TIME*Meth** | **4** | **1** | 0 | . | . | . | . |
| **TIME*Meth** | **4** | **2** | 0 | . | . | . | . |

| **Type 3 Tests of Fixed Effects** | | | | |
| --- | --- | --- | --- | --- |
| **Effect** | **Num DF** | **Den DF** | **F Value** | **Pr > F** |
| **TIME** | 1 | 17.1 | 3.00 | 0.1013 |
| **Meth** | 1 | 15.8 | 0.43 | 0.5201 |
| **TIME*Meth** | 1 | 22 | 0.00 | 0.9604 |

| **Least Squares Means** | | | | | | | | | | |
| --- | --- | --- | --- | --- | --- | --- | --- | --- | --- | --- |
| **Effect** | **TIME** | **Meth** | **Estimate** | **Standard Error** | **DF** | **t Value** | **Pr > \|t\|** | **Alpha** | **Lower** | **Upper** |
| **TIME*Meth** | **5** | **1** | 1.0377 | 0.03477 | 20.6 | 29.84 | <.0001 | 0.05 | 0.9653 | 1.1101 |
| **TIME*Meth** | **5** | **2** | 1.0058 | 0.06020 | 18.5 | 16.71 | <.0001 | 0.05 | 0.8796 | 1.1320 |
| **TIME*Meth** | **4** | **1** | 0.9600 | 0.04079 | 17.2 | 23.53 | <.0001 | 0.05 | 0.8740 | 1.0460 |
| **TIME*Meth** | **4** | **2** | 0.9247 | 0.06854 | 16.6 | 13.49 | <.0001 | 0.05 | 0.7798 | 1.0696 |


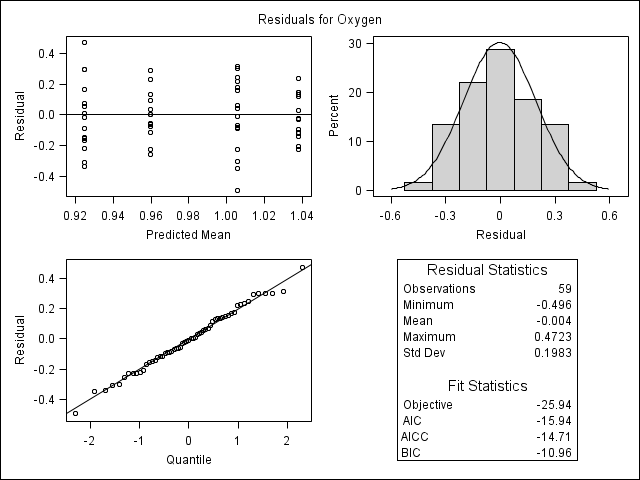


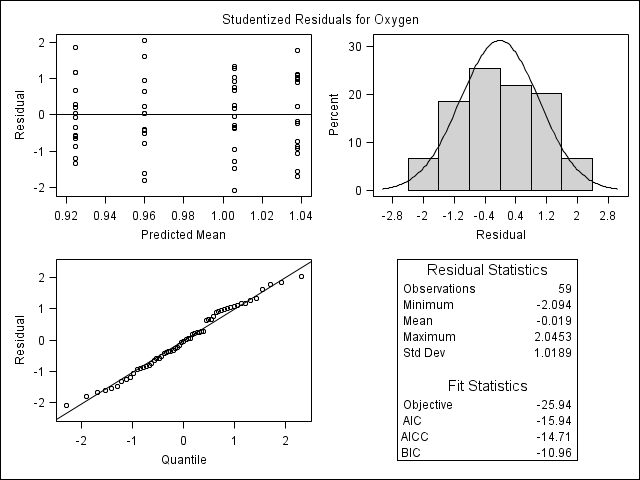


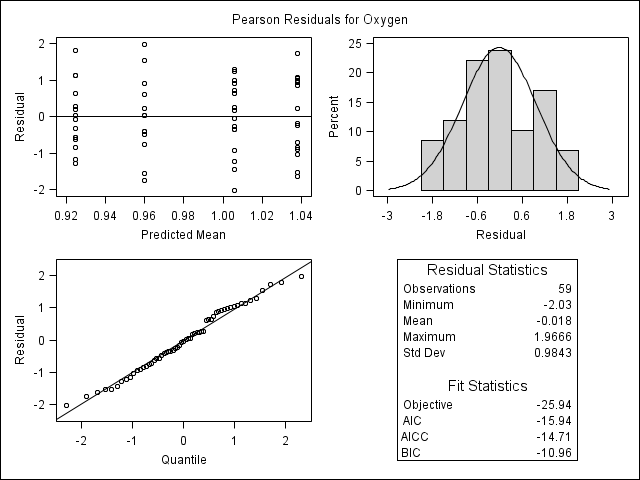

Supplement: S2 File — S2:1 Mixed effect models for parallelity analysis between GVO2 and EVO2 normalised to baseline Anaesthesia (T0) (= 1.0); Early surgery (T2); Late surgery (T3); Early postop (T4); Late postop (T5) pp. 2–9 S2:2. Mixed effect models for parallelity analysis between GVO2 and Ca-cvO2 (arterio-central venous oxygen content difference) normalised to baseline Anaesthesia (T0) (= 1.0); Early surgery (T2); Late surgery (T3); Early postop (T4); Late postop (T5) pp.10–17 S2:3 Mixed effect models for paralellity analysis between GVO2 and CI (cardiac index) normalised to baseline Anaesthesia (T0) (= 1.0); Early surgery (T2); Late surgery (T3); Early postop (T4); Late postop (T5) pp.18–25 S2:4.Mixed effect models for parallelity analysis between GVO2 and EVO2 normalised to overall mean for each method (= 1.0) in anaesthetized subjects Anaesthesia (T0); Early surgery (T2); Late surgery (T3) pp.26–31S2:5. Mixed effect models for paralellity analysis between GVO2 and EVO2 normalised to overall mean for each method (= 1.0) in awake subjects Early postop (T4); Late postop (T5) pp. 32–37. (DOCX) [file pone.0272239.s002.docx]
